# Redox Status in Canine Leishmaniasis

**DOI:** 10.3390/ani11010119

**Published:** 2021-01-08

**Authors:** Fausto Quintavalla, Giuseppina Basini, Simona Bussolati, Gennaro Giuseppe Carrozzo, Antonio Inglese, Roberto Ramoni

**Affiliations:** 1 Dipartimento di Scienze Medico-Veterinarie, Università degli Studi di Parma, Via del Taglio 10, 43126 Parma, Italy; basini@unipr.it (G.B.); simona.bussolati@unipr.it (S.B.); roberto.ramoni@unipr.it (R.R.); 2 Clinica Veterinaria Zoolife, Via Cavalleggeri Udine, 38017 Mezzolombardo (TN), Italy; gennaro.carrozzo@gmail.com; 3 Ambulatorio Veterinario, Via Friuli 19, 74023 Grottaglie (TA), Italy; inglese.antonio.vet@gmail.com

**Keywords:** dog, redox status, free radicals, scavenger enzymes, leishmaniasis

## Abstract

**Simple Summary:**

Leishmaniasis is under strict observation by World Health Organization but its pathogenesis has not been completely clarified yet. Our aim was to compare healthy and affected dogs measuring parameters related to oxidative stress, namely reactive oxygen species, reactive nitrogen species and scavenger activities, using colorimetric assays. Our results demonstrate that several of the examined parameters are modified in canine Leishmaniasis. Therefore, it is essential to further investigate this topic to shed light on the pathogenesis of the disease.

**Abstract:**

The World Health Organization defined leishmaniasis as one of the priority attention diseases. Aiming to clarify some aspects of its pathogenetic mechanisms, our study focused on the assessment of redox status in dogs, the main reservoir for *Leishmania infantum*. Forty-five dogs from an endemic area in southern Italy were divided into four different groups (from mild disease with negative to low positive antibody levels to very severe disease with medium to high positive antibody levels) according to the LeishVet group guidelines. Their plasma and/or sera were tested for reactive oxygen species (ROS), namely the superoxide anion (O_2_^−^), reactive nitrogen species (RNS), such as nitric oxide (NO) and hydroperoxides (ROOH), as well as activity of the detoxifying enzyme superoxide dismutase (SOD), and total nonenzymatic antioxidant capacity, as determined by the ferric reducing-antioxidant power (FRAP) assay. O_2_^−^ generation was significantly (*p* < 0.05) reduced in leishmaniasis-affected dogs independently of the clinical stage, while NO production was stimulated (*p* < 0.05) only in II and III stage patients. No difference could be found for the levels of hydroperoxides and SOD activity between healthy and pathological subjects. FRAP values were lower in affected dogs but only in stage II. Taken together, although we demonstrated that several redox status parameters are altered in the plasma of dog affected by leishmaniasis, the oxidative stress changes that are observed in this disease, are possibly mainly due to cellular blood components i.e., neutrophils responsible for the elimination of the parasite. Further studies are required to assess the clinical values of the collected data.

## 1. Introduction

Leishmaniasis is an infectious disease caused by parasites belonging to the genus *Leishmania* that are diffused by the puncture of sand flies of the genus *Phebotomus* spp., and *Lutzomyia* spp., in the Old and New World respectively [1]. Its potential lethality and worldwide diffusion motivated the World Health Organization (WHO) to consider leishmaniasis as one of the priority attention diseases [2]. Infection is caused by the injection of metacyclic promastigotes during blood-feeding, and the composition of the parasite dose appears to be crucial for the occurrence of the disease [3]. Although dogs are the main reservoir of the *Leishmania infantum*, cats and several other wild animals are thought to be involved in the diffusion of the disease in the Mediterranean countries [4]. The parasite is transmitted by vectors to its vertebrate host and then phagocytosed by macrophage-derived monocytes, where they survive and replicate [5]. The disease can present clinical forms classified as cutaneous leishmaniasis, mucocutaneous leishmaniasis, up to visceral leishmaniasis, that is the most severe [1]. In most of the cases, visceral leishmaniasis is concomitant with monocytic and hemolytic anemias [6]. High levels of lipid peroxidation are reported to be part of the pathogenic mechanisms of hemolytic anemia [7]. In the last few years, several authors have investigated the impact of oxygen free radicals on the pathogenesis of parasitosis both in humans [8,9,10,11,12] and in dogs [5,13,14,15,16,17,18,19,20]. Protozoa of the genus *Leishmania* are able to evade the immune system and perpetuate infection. The outcome of these investigations shows the infecting parasite initially behaves like a Trojan horse with respect to neutrophils by inhibiting their oxidative metabolism [21]. Using an experimental model, Daneshvar et al. [12] examined redox status in leishmaniasis with a proteomic approach and observed altered expression of a group of proteins which mediate response to reactive oxygen intermediates and confirmed that the attenuated *Leishmania* are more susceptible to oxidative challenge. Even if *Leishmania* parasites are damaged in vitro by the chemical activity of several reactive oxygen species (ROS; superoxide radical, hydrogen peroxide, nitric oxide and peroxynitrite), they can resist the endogenous oxidative burst that follows phagocytosis by mammalian macrophages [11]. However, the oxidative stress balance in leishmaniasis is far to be clarified. Therefore, the present study aimed to unravel redox status in canine leishmaniasis measuring ROS in plasma samples, namely superoxide anion (O_2_^−^), reactive nitrogen species (RNS) such as nitric oxide (NO) and hydroperoxides (ROOH), the activity of the detoxifying enzyme superoxide dismutase (SOD) and the total nonenzymatic antioxidant capacity, as determined by the ferric reducing-antioxidant power (FRAP) assay.

## 2. Materials and Methods 

### 2.1. Animals

This study was submitted to the Committee for Animal Ethics of the University of Parma (approval number PROT. 06B-CE20 04/02/20), and the experiments were realized following the approved guidelines. Forty-five owned dogs with clinical manifestation and laboratory abnormalities (electropherogram and biochemical profile) related to leishmaniasis and never subjected to pharmacological treatments were enrolled from an endemic area in the south of Italy (Taranto). Blood was collected from the cefalic vein. Serum was separated by centrifugation within 15 min of collection. Plasma (lithium heparin) was collected and stored in cryovials (−80 °C) until redox status analyses were performed. All the dogs to be included in this study had to be negative for the in vitro serum tests for the detection of the *Dirofilaria immitis* antigen and for the antibodies against *Anaplasma phagocytophilum*, *Anaplasma platys*, *Borrelia burgdorferi*, *Ehrlichia canis* and *Ehrlichia ewingii* (SNAP 4D* Plus, Iddex Laboratories—Hoofddorp NL). Serum was assayed by ELISA (enzyme-linked immunosorbent assay) kit for the diagnosis of canine leishmaniasis (Leishmania Ab ELISA Biopronix Agrolabo S.p.A., Turin, Italy). Thereafter, all the samples with a positive result were subjected to the immunofluorescence antibody test (IFAT), and staged in four different groups in agreement with the Manual of Standards for Diagnostic Test and Vaccines proposed by the LeishVet group [22]. In particular, eight dogs were included in Stage I (antibody level 1:40–1:80) 9 dogs in Stage II (1:160–1:320), 11 dogs in Stage III (1:640–1:1280) and 17 dogs in Stage IV ( ≥1:2560). Twenty-four owned healthy dogs (Table 1), without clinical signs of leishmaniasis, all free from haemoparasitosis and with a negative outcome in the serological leishmania test, were enrolled as control from the nonendemic area (Parma). All the dogs enrolled in the present investigation had not been vaccinated against *Leishmania*.

### 2.2. Measurement of Reactive Oxygen Species (ROS) and Reactive Nitrogen Species (RNS)

All the reagents used were from Sigma Chemical Co Lt (St. Louis, MO, USA), except those specifically mentioned.

#### 2.2.1. Assay for Superoxide Anion (O_2_^−^)

Since evidence exists that tetrazolium salts can be used as a reliable measure of O_2_^−^ production, this parameter was evaluated by the WST-1(Water-soluble tetrazolium salt-1) test (Sigma Chemical Co Lt, St. Louis, MO, USA). Briefly, 25 μL of plasma, 75 μL of PBS and 10 μL of WST-1 reagent were added to each well of a 96-well plate and incubated for 4 h at 37 °C. The absorbance of each sample was then determined using the reader Multilabel Counter Victor3 (Perkin Elmer, Boston, MA, USA) at 450 nm from which was subtracted the absorbance value at 620 nm [23].

#### 2.2.2. Assay for Nitric Oxide (NO)

NO was assessed in plasma by measuring nitrite levels with a method based on the formation of a chromophoric compound after reaction with the Griess reagent, that was prepared fresh by mixing equal volumes of stock A (1% sulfanilamide, 5% phosphoric acid) and stock B (0.1% N-[naphthyl] ethylenediamine dihydrochloride) solutions. A calibration curve ranging from 0.39 to 25 μΜ was prepared by diluting a water solution of sodium nitrite in distilled H_2_O. The assay was performed in 96-well plates by adding 30 µL of serum to 70 µL of distilled water and 50 µL Griess reagent. After incubation, the absorbance was determined with the Multilabel Counter Victor 3 (Perkin Elmer, Boston, MA, USA) at 540 nm from which was subtracted the absorbance value at 620 nm [24].

#### 2.2.3. Assay for Hydroperoxides (ROOH)

The so called Diacron reactive oxygen metabolites were quantified in plasma by using a d-ROM test purchased from Diacron s.r.l. Grosseto, Italy. The test is based on the reaction that occurs between hydroperoxides and the iron released from the endogenous proteins in consequence of the acidic pH of the R2 reagent of the kit(Diacron s.r.l. Grosseto, Italy), that following Fenton reaction mechanism give raise to peroxyl and alkoxyl radicals (ROOH). These compounds finally develop a pink color in the R1 reagent of the test through the reaction with an alkyl-substituted aromatic amine. Briefly, 2 µL of chromogenic substrate (R1) and 200 µL of buffer, pH 4.8 (R2), were mixed with 2 µL of plasma in each well of a microplate. A blank reagent, obtained by replacing the plasma with distilled water and a standard calibrator sample containing known amounts of ROOH (provided by the manufacturer, Diacron s.r.l. Grosseto, Italy), were included for each assay. After 20 min of incubation at 37 °C, the absorbance was measured at 540 nm by Multilabel Counter Victor3 (Perkin Elmer, Boston, MA, USA). The results were expressed in arbitrary units called Carratelli Units (CARR U) according to the following formula:

$$\mathrm{CARR}\;U=\mathrm{Absorbance}\;\mathrm{sample}÷\mathrm{Absorbance}\;\mathrm{calibrator}\times \left[\mathrm{calibrator}\right]$$

as indicated by the manufacturer [25].

### 2.3. Scavenging Enzyme Activity

#### 2.3.1. Assay for Superoxide Dismutase (SOD)

SOD levels in plasma were assessed by using a commercial enzymatic activity assay (Sigma Chemical Co Lt, St. Louis, MO, USA. The enzyme activity was quantified by measuring the amount of formazan produced by the reaction between tetrazolium salt (WST-1) and superoxide anion (O_2_^−^), that is generated by the reaction of an exogenous xantine oxidase. The remaining O_2_^−^ is an indirect hint of the endogenous SOD activity. In each well of a microplate, 20 µL of plasma were added with 200 µL of WST working solution and 20 µL of enzyme working solution. A standard curve of SOD ranging from 0.1 to 200 U/mL was prepared. The color intensity was determined with Multilabel Counter Victor3 by measuring the absorbance at 450 nm from which was subtracted the absorbance value at 620 nm [26].

#### 2.3.2. Scavenging Nonenzymatic Activity: Ferric Reducing-Antioxidant Power (FRAP)

The reducing ability of the plasma samples was determined by the so called FRAP assay. The FRAP assay measures the change in absorbance at 620 nm due to the formation of a blue colored Fe^++^-tripyridyltriazine (TPTZ) compound from colorless oxidized Fe^+++^ form by the action of electron donating antioxidants. Briefly, 20 µL of plasma were mixed with 20 µL of distilled water and with 260 µL of FRAP reagent in each well of a 96-well plate. FRAP reagent was prepared fresh by mixing 25 mL acetate buffer (0.3 M; pH 3.6), 2.5 mL TPTZ (10 mM in 40 mM HCl), and 2.5 mL FeCl_3_•6H_2_O (20 mM). Aqueous solutions of known Fe++ (FeSO4•7H_2_O) concentration in the 100–1000 µM range were used for the calibration curve. The absorbance was recorded with Multilabel Counter Victor3 (Perkin Elmer, Boston, MA, USA) at 620 nm after a 30-min incubation at 37 °C [27].

### 2.4. Statistical Analysis

Experimental data are presented as mean ± SEM; statistical differences were calculated with ANOVA using Statgraphics package (STSC Inc., Rockville, MD, USA). When significant differences were found, means were compared by Scheffè’s F test.

## 3. Results

The average age of dogs with leishmaniasis was four years of which 26 were males, 19 females of which four were sterilized. The average body weight was 22.8 kg. In addition to the mongrel (12), the most represented dog breeds were German Shepherd (7) and Epagneul Breton (5). In the control group, on the other hand, the average age was 5,7 years and the majority of dogs were males (15 vs. 9), in the majority of cases mongrel (16) (Table 2).

### 3.1. Reactive Oxygen Species (ROS)

**Figure 1:**
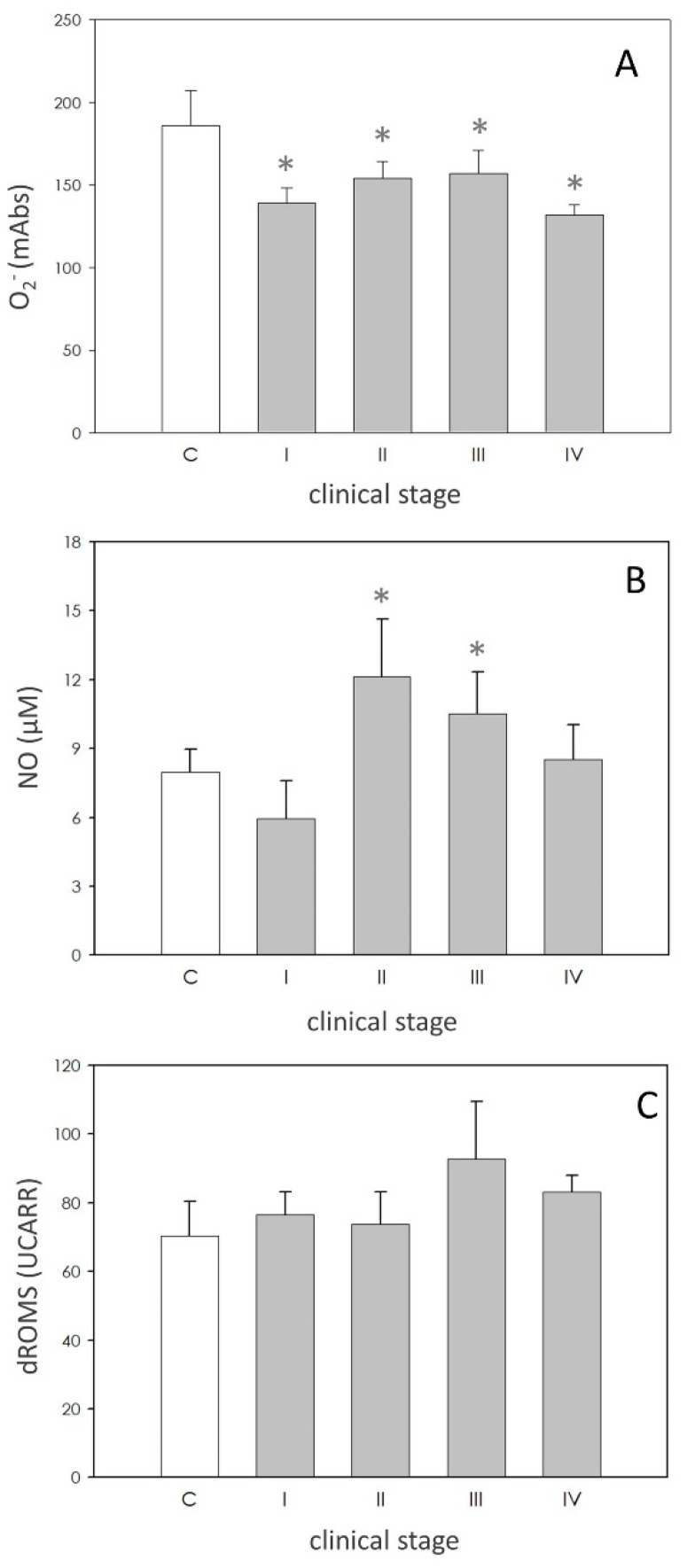
Reactive oxygen species (ROS) and reactive nitrogen species (RNS). Panel (**A**): superoxide anion (O_2_^−^) levels expressed as milliabsorbance units (mAbs). Panel (**B**): nitic oxide (NO) expressed as µM. Panel (**C**): hydroperoxide levels expressed as Carratelli Units (UCARR). In each panel, asterisk (*) on the bar indicate significant difference (*p* < 0.05) as assessed by ANOVA and Scheffè F test.

O_2_^−^ levels were significantly (*p* < 0.05) reduced in the dogs affected by leishmaniasis; nevertheless, the values of this parameter didn’t show any significant differences between different stages (Figure 1A).

Compared to the data of the subjects of the control group, NO production was stimulated (*p* < 0.05), approximately at the same level, in dogs in II and III stages, while it was unaffected in those of stage I and IV (Figure 1B).

As revealed by the dROMS test, no difference could be found for the levels of hydroperoxides between healthy and pathological subjects (Figure 1C).

### 3.2. Scavenging Activity of ROS

#### 3.2.1. Superoxide Dismutase (SOD)

**Figure 2:**
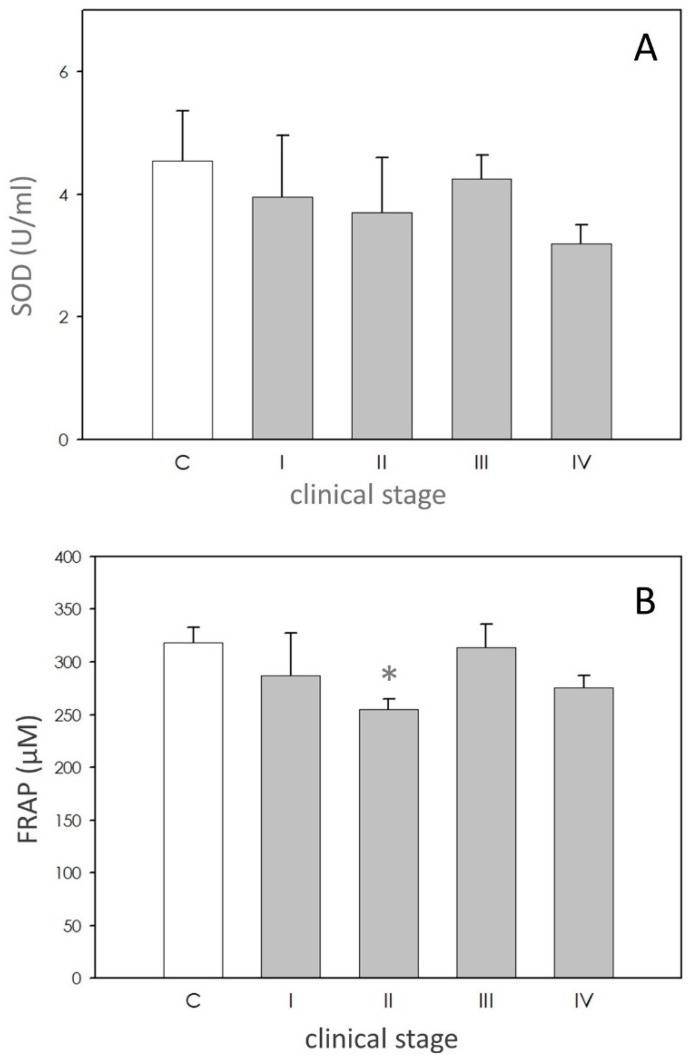
Scavenging activity. Panel (**A**): superoxide dismutase (SOD) activity expressed as units/mL (U/mL). Panel (**B**) ferric reducing-antioxidant power (FRAP) levels expressed as µM. In each panel, asterisk (*) on the bar indicate significant difference (*p* < 0.05) as assessed by ANOVA and Scheffè F test.

SOD activity in dogs affected by leishmaniasis exhibited approximately the same level of the controls (Figure 2A).

#### 3.2.2. Scavenging Nonenzymatic activity: Ferric Reducing-Antioxidant Power (FRAP)

FRAP values were lower in affected dogs, but only in stage II (Figure 2B).

## 4. Discussion

In Mediterranean leishmaniasis endemic regions, seroprevalence varies from 2 to 40%. Nevertheless, the exposure rate of dogs to the parasite is probably much higher [14], also given climate change in act. Moreover, recent research indicates a widespread distribution of the disease also in northern Italy [28]. Canine leishmaniasis (CL) has a bimodal distribution, with a peak in subjects less than three years old, and a second peak between eight and 10 years [29]. In our study the average age of the animals enrolled, that were naturally infected by *Leishmania infantum* was higher than expected (four years). This may be due to the fact that CL presents a widespread range of clinical manifestations varying from visceral to cutaneous or viscerocutaneous presentation and polymorphic clinical aspects in times ranging from a few weeks to many months, thus determining a delay before the owner submits the pet to a clinical visit, as recently observed by Pereira et al. [30]. Except for the animals of stages I and II, in the present study male dogs >2 years with medium to high positive antibody levels were the most predisposed, thus suggesting that gender can be involved in the development of the disease, as already shown by other research [31]. The literature indicates that leishmaniasis causes increased oxidative stress in canine neutrophils, with an intensity dependent on the stage of the disease [32]. Symptomatic dogs show more severe oxidative stress than less symptomatic and noninfected dogs. Sousa and coworkers stated that animals exhibiting evident canine visceral leishmaniasis (CVL) symptoms show low levels of antioxidant enzymes [19]. Moreover, enhanced lipid peroxidation may be linked to liver and kidney damage in CVL [14,16]. In particular, BUN (Blood Urea Nitrogen) and creatinine concentrations are the major biochemical findings of leishmaniasis, and the variability and degree of biochemical abnormalities depend mainly on progression of the disease [15], assuming also a prognostic value [30]. Conversely, the decrease of almost all the reducing capacities of the organism may be a result of the host’s defense mechanisms against the increase in oxidation caused by the parasite. An enhanced ROS and RNS production by the organism are considered defense strategies that may amplify the leishmanicidal activity in human patients with cutaneous leishmaniasis. However, these intermediates not only cause killing of the parasite but also may cause DNA damage to the adjacent cells, possibly leading to cancer development [9]. Similar observations were reported from Serarsalan et al. [10] who observed significantly higher levels of serum malondialdehyde (MDA). ROS overproduction can induce an imbalance between oxidant and antioxidant at the cellular or systemic level, leading to the establishment of oxidative stress [33]. O_2_^−^ is the precursor of other harmful oxidants such as H_2_O_2_ and its derivates [11], and O_2_^−^ production was significantly elevated during phagocytosis of the stationary phase promastigotes [34]. The significant reduction of O_2_^−^ in affected dogs, independent of the clinical stage, is a strong result in our study. This finding let us hypothesize that the parasitocidal mechanism of peripheral blood monocytes towards leishmania in the affected dogs were weakened as already suggested [35].

Nitric oxide (NO) has been demonstrated to be the molecule mostly involved in intracellular killing of *Leishmania*, both in vitro and in vivo [36]. In dogs, single and coinfections by *L. infantum*, *E. canis* and *B. vogeli* cause an increase in the levels of NO [33]. NO becomes increasingly important as a defense mechanism during the intracellular amastigote stage [11]. In this study we found a significant increase of NO levels in plasma of patients of stages II and III, thus confirming the involvement of this nitrogen radical in the pathogenesis of the disease. In the present study, focused on plasma, variation of the values of dROMS was not observed except for the animals in stages III and IV, thus suggesting that the imbalance of the markers possibly involves mainly neutrophils, which are the host cells mostly involved in the elimination of the parasite. A significant increase of SOD values was reported in plasma of a leishmaniotic group of dogs [35] in agreement with results reported for human patients with cutaneous leishmaniasis [10,37,38], but in contrast with observations of other authors [39,40]. In our samples, SOD activity was not modified. However, dogs with the highest clinical score displayed lower, even not significant, levels of this enzyme, possibly reflecting exhaustion of the immune system [41]. FRAP measures were not different between healthy and leishmaniotic dog in the study of Rubio et al. [36,41] while another research showed a significant reduction in FRAP levels in affected symptomatic dogs. Our data point out the reduction in FRAP only in the dogs of stage II. We hypothesize that the weakening in antioxidant power could represent a critical point in the development of the disease. This finding deserves further research.

## 5. Conclusions

The imbalance of oxidants and antioxidants has been demonstrated in several animal species and in many pathological conditions including parasitic diseases. The present investigation demonstrates redox status imbalance in leishamaniasis, since O_2_^−^ generation was reduced while NO production and FRAP values were modified depending on the clinical stages. Hydroperoxides and SOD activity were unaffected. Taken together, we argue an involvement of those blood cells that are responsible for the elimination of the parasite. Aware of the fact that a clinical case should be implemented, we advise that the therapeutic approach of canine leishmaniasis could require a more adequate treatment in light of the patient’s redox state.

## Figures and Tables

**Table 1:** The table reports the data (age, sex, weight) of the belonging to the control group.

Dog	Breed	Age (Years)	Sex
1	Mongrel	4	F
2	Mongrel	10	F
3	Beagle	7	F
4	Mongrel	5	F
5	Beagle	4	M
6	Beagle	9	M
7	English setter	3	M
8	Mongrel	4	M
9	Mongrel	3.5	M
10	Mongrel	6	M
11	Mongrel	5	M
12	Mongrel	4	M
13	Poddle, Miniature	9	M
14	Mongrel	4	M
15	Mongrel	3	F
16	Mongrel	7.5	F
17	Mongrel	9	F
18	Mongrel	4	F
19	Riesenschnauzer	5	F
20	Mongrel	6	M
21	Mongrel	3	M
22	Mongrel	4	M
23	Dachshund	10.5	M
24	Labrador retriever	8	M

F = female, M = male.

**Table 2:** The table reports the data (age, sex, weight) of the dogs divided into four categories according to the classification proposed by the LeishVet group.

	Breed	Age (Years)	Sex	BW (kg)
**Stage I** mild disease with negative to low positive antibody levels	Epagneul Breton	5	F	14
	German Shepherd dog	3.5	M	46
	Mongrel	6	Fs	12
	Mongrel	5	M	12
	Mongrel	5	M	23
	Mongrel	6	F	26
	Dachshund	10	M	12
	Pug	2	F	10
**Stage II** moderate disease with low to high positive antibody levels	Pug	3	F	9
	German Shepherd dog	4	F	40
	Mongrel	3.5	F	8
	English Pointer	3	F	18
	English Cocker Spaniel	6.5	M	14
	Mongrel	1.3	F	9
	Bernese mountain dog	7	M	37
	Epagneul Breton	4.6	F	17
	French Bulldog	4.8	M	13
**Stage III** severe disease with medium to high positive antibody levels	Dogo Argentino	2	M	30
	German Shepherd dog	4	M	27
	Boxer	3	F	27
	Pinscher	6	M	3.2
	Mongrel	3	M	17
	Epagneul Breton	2.5	M	13
	Mongrel	4	F	12
	Epagneul Breton	3	Fs	32
	Mongrel	8.5	M	26
	Bracco italiano	1.5	M	26
	Mongrel	5.5	F	32
**Stage IV** very severe disease with medium to high positive antibody levels	Labrador retriever	3	M	38
	American Staffordshire terrier	1	M	23
	Pinscher	3	M	6
	Pinscher	5	M	3.5
	German Shepherd dog	5	M	36
	Epagneul Breton	2.5	F	11
	Irish Setter	4.5	M	29
	St Bernard dog	3	F	67
	Mongrel	5	M	22
	Cane da Pastore maremmano abruzzese	4	Fs	44
	German Shepherd dog	1.5	M	36
	Mongrel	7	Fs	25
	German Shepherd dog	2	M	39
	German Shepherd dog	3.5	M	31
	Shar Pei	3	M	12
	Jack Russell terrier	3	F	8.5
	Mongrel	5	M	30

F = female, Fs = female sterilized, M = male.

## Data Availability

04/02/20.
